# A meta‐analysis: *microRNAs’* prognostic function in patients with nonsmall cell lung cancer

**DOI:** 10.1002/cam4.1158

**Published:** 2017-08-15

**Authors:** Na Yu, Qingjun Zhang, Qing Liu, Jiayu Yang, Sheng Zhang

**Affiliations:** ^1^ Department of Epidemiology and Medical Statistics Wuhan University Hubei China; ^2^ The Center for Disease Control and Prevention of Hubei Province Hubei China; ^3^ Zhongnan Hospital of Wuhan University Hubei China; ^4^ Department of Epidemiology and Medical Statistics Nantong University Jiangsu China

**Keywords:** Meta‐analysis, *miRNA*, nonsmall cell lung cancer, overall survival

## Abstract

Accumulating papers have demonstrated that microRNAs play an important role in the progression of lung cancer, mainly as oncogenic and tumor suppressive. Therefore, microRNAs may influence the survival of lung cancer patients. In this meta‐analysis, we evaluated the role of *microRNAs* in affecting the overall survival in nonsmall cell lung cancer (*NSCLC*) patients, which may provide valuable information for the treatment of nonsmall cell lung cancer. We used keywords to retrieve literatures from online databases *PUBMED*,*EMBASE* and Web of Science and included 12 studies into our investigation according to pre‐set criteria. Then, we analyzed the data with stata13.1 to evaluate the microRNAs role on the prognosis of *NSCLC* patients. *NSCLC* patients with higher microRNAs expression levels tend to show lower overall survival. HR (hazard ratio): 2.49, 95% CI (confidence interval): 1.84–3.37. Besides, both oncogenic and tumor suppressive microRNAs have an evident influence on prognosis with HR values of 2.60 (95% CI: 2.12–3.19) and 0.41 (95% CI: 0.05–0.34), respectively. *microRNAs*, especially from tissue, have an influence on overall survival of *NSCLC* patients, which indicates that microRNAs could serve as potential prognostic markers for *NSCLC* and may provide a treatment strategy for advanced *NSCLC* patients.

## Introduction

Lung cancer is the number one cause of cancer‐related mortality in both men and women, and approximately 80% is nonsmall cell lung cancer (*NSCLC*) 8. Despite recent advances in the diagnosis and chemotherapeutic and targeted treatment of *NSCLC*, including immunotherapy, such as epidermal growth factor receptor (*EGFR*)‐targeted treatment, insulin‐like growth factor 1 receptor or *EML4‐ALK* fusion protein interference 7, the overall survival rate of *NSCLC* patients remains low (5‐year survival rate of 15%) and the recurrence rate of *NSCLC* remains high, even with early diagnosis 25. MicroRNAs are small, noncoding, RNA molecules that regulate gene expression typically by binding the 39 untranslated region (*UTR*) of *mRNA* 16. In several biological processes, such as cell proliferation, differentiation, migration and apoptosis, *microRNAs* are involved in regulating the expression of multiple target genes 5, 17, 20. The clinical usefulness of *miRNA* expression analysis to predict the efficacy of various treatment strategies including surgery, radio‐ and chemotherapy, and targeted therapies has been evaluated in *NSCLC* 3. A similar research about *microRNAs*’ prognostic function in breast cancer patients has been published 14. This meta‐analysis aimed at analyses‐related studies to produce a reliable outcome on whether *microRNAs* are credible prognostic biomarkers for patients with *NSCLC*.

## Materials and Methods

### Literature retrieval strategy

The studies were retrieved by two reviewers from online databases *PUBMED*,* EMBASE* and Web of Science. We selected the English literatures carried out on human subject and publication before March 31, 2017. The key words for the literature retrieval strategy included “*microRNA*,” “*miRNA*,” “nonsmall cell lung cancer,” “*NSCLC,*” “*prognos*,*” “*survi*,*” “Kaplan–Meier,” and “HR”. The search was further restricted to English‐language articles and human subjects. All references from eligible publications in the literature were screened manually for further potential literature (Table S1–S3).

### Criteria for inclusion and exclusion

Included studies met the following criteria: (1) enrolled research subjects being *NSCLC* patients with healthy or normal individual as control; (2) investigation of the association between *miRNA* expression levels and overall survival of the *NSCLC* patients. Literatures were excluded if they had one or more of the following criteria: (1) tissues or materials were from animals instead of human; (2) the study focus on other types of cancers instead of *NSCLC* only; (3) absence of survival outcomes or reported outcome could not be calculated; (4) overviews, reviews, symposium papers, comments, reports, letters, and duplicate publications are excluded.

If two or more trials with different outcomes, such as HR, 95% CI, were carried out in the same article, or the corresponding outcomes could be calculated by Kaplan–Meier curves, we recognized them as two or more independent publications. When univariate and multivariate analysis were carried out at the same time, we chose the latter as the final outcome of the corresponding factor, which should be treated as the more precise result. Besides, among different publications that investigated the same cohort patients, we chose the most completed research.

### Quality assessment and data extraction

Two reviewers evaluated the quality of the enrolled studies independently using the guideline of the Newcastle‐Ottawa Quality Assessment Scale (*NOS*) 21, and each study was marked with scores ranging from 0 to 9. After evaluation, the researches with a score greater than 6 was considered as high quality.

The following data are extracted from all included publications by two reviewers independently:name of the first author, year of publication, country and area, numbers of the research objects, sample source, type of *miRNA*(s), treatment to patients, cutoff value, follow‐up time (basic unit: month), HR values, 95% CI and *P* value of *microRNAs* for predicting overall survival (*OS*) and disease‐free survival (*DFS*). For the literatures that did not report any HR values and 95% CI shown in some literatures, we calculated the values using provided Kaplan–Meier curves and related statistical methods 24. After the calculation, we obtained HR values in ten articles and *RR* (relative risk) values in two articles. We collected all HR values based on high versus low expressions of *miRNA*. As for follow‐up time, we got them from the original articles or Kaplan–Meier curves.

### Statistical analysis

STATA 13.1 was utilized for this meta‐analysis. All provided HR values and their corresponding 95% CI, shown as high versus low, were used as original data to study the collected prognostic value of the *OS* and *DFS* (disease‐free survival) of *NSCLC*, while for unknown HRs, we obtained the Kaplan–Meier curves from the original papers and chose 33 points in each graph to get 33 corresponding X and Y values for calculation 24. In total, we calculated three times independently for each publication and chose the medium as the final value. Evidently, *NSCLC* patients with poor prognosis tend to have an overexpression *microRNAs* with pooled HR values over one. Heterogeneity among the studies was evaluated by Cochran's Q test and Higgins's *I* ^2^ statistics. Heterogeneity was taken into account when *P* < 0.10 and *I* ^2^ > 50%, so we analyzed the data firstly in the random‐effect model to examine whether *I* ^2^ was over 50%, and then chose the appropriate model to investigate its heterogeneity. We carried out this meta‐analysis mainly based on different resources of *microRNAs*, oncogenic, and tumor suppressive *microRNAs* in patients, follow‐up time and different treatment of enrolled subjects. Publication bias was described by Egger's and Begger's bias test.

## Results

### The quality of enrolled studies

The *NOS* was used to assess the quality of the 12 studies 2, 4, 9, 12, 13, 15, 18, 26, 28, 29, 30, 31 included in the meta‐analysis, and we gave the responding scores according to its meeting items (Table S4). As reported, these studies were cohort studies and they aimed to get survival outcome in the exposure of disease or not, as well as the expression level of *microRNAs*. Thankfully, all of them had suitable controls that controlled any known factors that may influence the outcome. In addition, they followed up the patients in considerable time and in ways that made the survival outcome convincible. Overall, the enrolled studies are considered as high quality with scores over 6, and high accuracy for our meta‐analysis.

### Literature search and study characteristics

One thousand and eight hundred potential literatures were found according to keywords searching in *Web of Science, PUBMED* and *EMBASE*. After deleting duplicate, unrelated essays, 675 literatures remained. Title and abstract screening was carried out first, and 542 articles were removed. With the guidance of exclusive and inclusive criteria, we subsequently chose the most relevant 12 publications including 22 *microRNAs* analysis, although 5 same *microRNAs* studied in 11 different trials, for this meta‐analysis after a full text reading. The excluded literatures lacked overall survival analysis or had analysis but did not present HR values that also could not be calculated. The inclusion and exclusion flowchart was shown in Figure S1.

The basic traits and information of the enrolled studies are shown in Table 1 and Table S5, respectively. The results of the subgroup analysis are shown in Table 2. Almost all the investigation detected the expression level of *microRNAs* in tissue or serum by *RT‐PCR*. The cutoff values for the expression were different. All of the literatures analyzed the correlation between microRNAs and OS and one of them also explained the association between expression level of microRNAs and *DFS* in *NSCLC* patients. The inclusive articles provided Kaplan–Meier curves directly, although only 8 of them included the HR values and 2 with *RR* values. We calculated the HR *s* and 95% CIs for the other 2 articles. Furthermore, we assessed the quality for each included study and the medium *NOS* score is 7, which means the inclusive article are in a high quality.

**Table 1:** Characteristics of the studies included in this meta‐analysis

Study ID	Country	Patients	Control	Sample	miRNA	Treatment	Survival analysis	Cutoff point	Follow‐up time (month)	HR values	Quality score	Ref
Liu 2017	Chongqing, China	196	10	Plasma	miR‐23b‐3p/ miR‐21‐5p/ miR‐10b‐5p	–	OS	–	3.43–36.87	Reported	7	12
Petriella 2016	Bari, Italy	30	10	Serum	miR‐486‐5p	Chemotherapy	OS	Median	<20	Reported	8	13
Zhao et al. 30.	Henan, China	80	60	Serum	miR‐21	None	OS	1.22	12–48	Calculated	7	14
Wu et al. 28	Fujian, China	94	94	Serum	miR‐19b miR‐146a	Chemotherapy	OS	–	<45	Calculated	7	15
Cui 2013	Shanghai, China	260	260	Serum	miR‐125b	Chemotherapy	OS	Mean	20	Reported	8	16
Liu 2012	Zhejiang, China	70	40	Tissue serum	miR‐200c miR‐21	Surgery	OS	2	24	Reported	6	17
Zhu et al. 31.	Zhejiang, China	70	44	Tissue serum	miR‐96 miR‐182 miR‐183	Surgery	OS	‐	<30	Reported	8	18
Wang et al. 26	Jiangsu, China	88	17	Serum	miR‐21	Surgery	OS	5fold	72	Reported	7	19
Kim et al. 9	SouthKorea	72	30	Tissue	miR‐126 miR‐200c	Surgery	OS	–	1–135	Reported	7	20
Guo et al. 4	Shanghai, China	25	25	Plasma	miR‐204	None	OS/DFS	0.023	<60	Reported	7	21
Mo et al., 15	Nanjing, China	73	53	Serum	miR‐1290	Surgery	OS	Median	60	Reported	7	22
Yang et al. 29	Beijing, China	74	52	PBMC	miR‐10b	None	OS	1.15	60	Reported	8	23

HR, hazard ratio; OS, overall survival; DFS, disease‐free survival.

**Table 2:** The results of the subgroup analysis

Subgroup	N	HR	LL	UL	*P*	*I* ^2^	*P* for heterogeneity
Total	22	2.491	1.841	3.370	0.000	66.40%	0.000
microRNAs resources							
Plasma	4	2.086	1.601	2.717	0.000	0.0%	0.810
Serum	11	1.873	1.154	3.041	0.011	75.6%	0.000
Tissue	6	4.623	2.705	7.902	0.000	0.0%	0.550
PBMCs	1	15.848	3.888	64.594	0.000.		0.000
Treatment							
Mixed	3	2.252	1.649	3.074	0.000	0.0%	0.000
Chemotherapy	4	0.673	0.151	2.991	0.603	90.1%	2.019
None	3	3.254	1.279	8.278	0.013	76.9%	0.4963
Surgery	12	3.222	2.252	4.612	0.000	34.8	0.1099
Follow‐up time							
<5 years	16	2.550	1.641	3.962	0.000	71.0%	0.4851
≥5 years	6	2.257	1.566	3.253	0.074	50.1%	0.0905

LL, lower limit; UL, upper limit; PBMCs, peripheral blood monouclear cells.

### Meta‐analysis of *miRNA*(s) in influencing the prognosis of *NSCLC* patients

**Figure 1:**
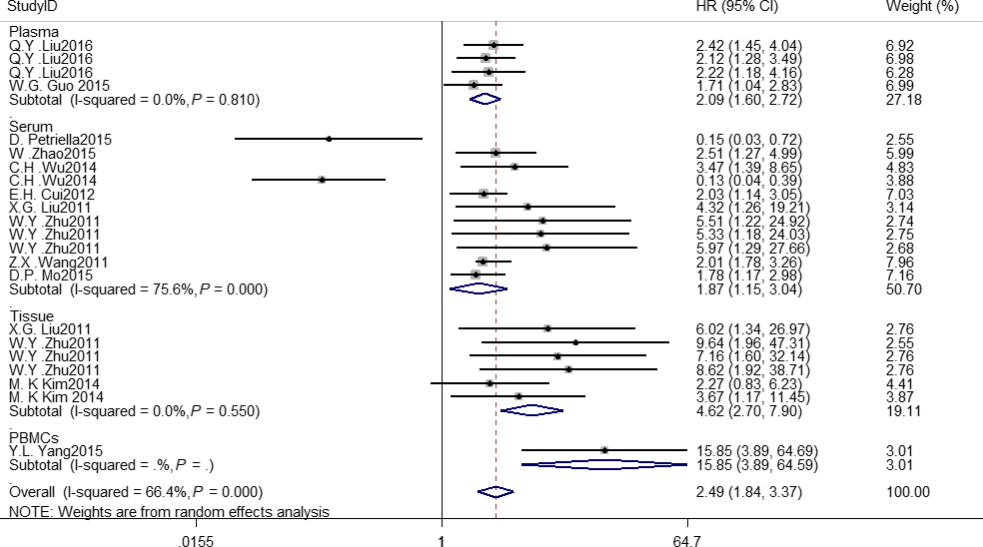
Meta‐analysis of subtotal HRs based on different resources of microRNAs.

**Figure 2:**
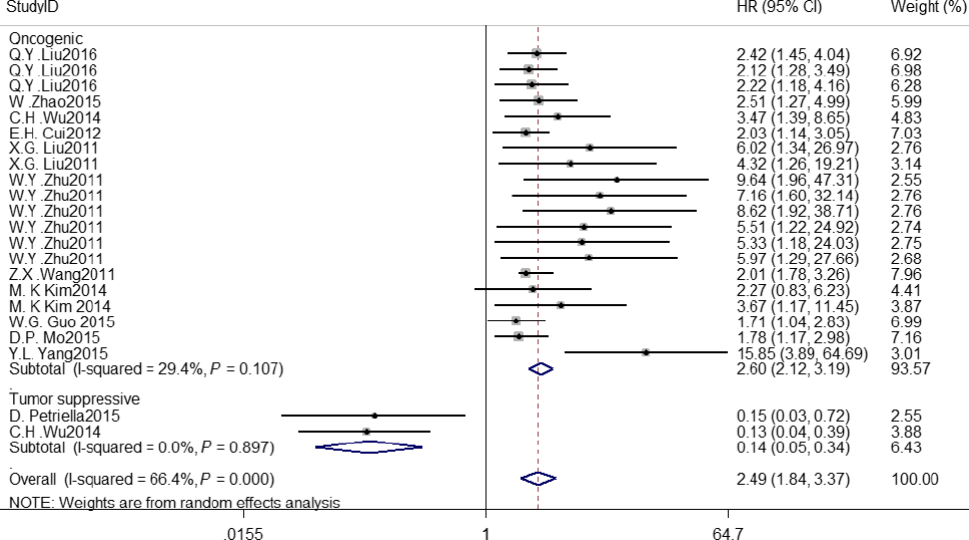
Meta‐analysis of subtotal HRs based on different function in effecting the OS of NSCLC patients.

The meta‐analysis was conducted to study the effect of total *microRNAs* in the prognosis of *NSCLC* patients, and the pooled HR of different resources of *microRNAs* are: plasma, 2.09 (95% CI: 1.60–2.72, *I* ^2 ^= 0.0%, *P* = 0.81); serum, 1.87 (95% CI: 1.15–3.04, *I* ^2 ^= 75.6%, *P* = 0.00); tissue, 4.62 (95% CI: 2.70–7.90, *I* ^2 ^= 0.0%, *P* = 0.55) (Fig. 1). Oncogenic and tumor suppressive *microRNAs* were analyzed to obtain their HRs values (Fig. 2). The pooled HR o are 2.60 (95% CI: 2.12–3.19) and 0.14 (95% CI: 0.05–0.34), respectively. By observation, we found that different follow‐up time for patients showed *microRNAs* contributed to a different overall survival (HR: 2.55, 95% CI: 1.64–3.96; HR: 2.26, 95% CI: 1.57–3.25) (Fig. S2). As for the treatment, surgery's HR is 3.22(95% CI: 2.25–4.61) with a low heterogeneity (*I* ^2 ^= 34.8%, *P* = 0.112), mixed treatment's HR is 2.25 (95% CI: 1.65–3.09) and chemotherapy's HR is 0.67 (95% CI: 0.15–2.91) (Fig. S3). Only one article included *DFS*, we chose to ignore this observation.

### Sensitivity analysis

**Figure 3:**
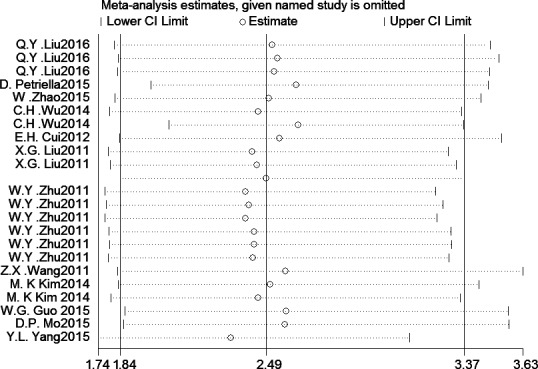
Sensitive analysis of meta‐analysis for microRNAs in the prediction of OS

A significant heterogeneity was observed in the comprehensive meta‐analysis even though the subgroups of oncogenic and tumor suppressive *microRNAs* showed a pretty low heterogeneity. Therefore, a sensitivity analysis should be performed to explore the source of the heterogeneity. (Fig 3).

### Publication bias

Publication bias existed in the included studies was examined by Egger's regression tests. The *P* value for egger plot was 0.163, and the Begger and Egger plots for the *OS* meta‐analysis and non‐*OS* meta‐analysis are shown in Figures S4 and S5, which suggest that no publication bias existed in this meta‐analysis.

## Discussion

Recent studies of microRNAs in lung cancer are summarized, focusing on microRNAs as diagnostic and therapeutic tools 22. Despite of recent progress in the understanding of the *miRNA* roles and their mechanism of function in biological pathways, there are still many obstacles to overcome prior to *microRNAs* technology entering the clinic. These obstacles include *microRNA* drug delivery, stability and tissue specificity of the therapeutic agent 6. Besides, the clinical utility of many reported microarray‐based prognostic gene signatures in lung cancer is questionable 23. Better understanding of the tumor molecular background is not only imperative for prescribing the most effective treatment for lung cancer, but can also be beneficial in risk assessment, disease diagnosis at earlier stages, more accurate classification of tumor type and predicting recurrence probability and treatment outcome. To date, differential gene expression is a well‐recognized platform for molecular profiling of lung tumors 1, 10, 11, 19, 27.

In this meta‐analysis, we revealed that high *miRNA* expression level was associated with a lower overall survival for *NSCLC* patients with HR values over one. Of course, we can see from the Figures 1 and 2 that significant heterogeneity was found in this study. We explored its heterogeneity by omitting each single study individually and re‐pooling the HRs of the remaining studies. No specific study influenced the overall HR values. MicroRNAs act as different function in the progression of *NSCLC*, so we divided the selected articles into subgroups based on oncogenes and tumor suppressors. They presented different subtotal HR values, respectively, and heterogeneity was discerned in the subgroups. This may explain the source of heterogeneity for the meta‐analysis. The HR of tumor suppressive microRNAs was significantly lower than that of oncogenic microRNAs, suggesting a better *OS* for *NSCLC* patients with high expression of tumor suppressive *microRNAs* and low expression of oncogenic *microRNAs*. In addition, different sources of *microRNAs* lead to a different *OS* compared to each other, then we can conclude that *microRNAs* from tissue could be stronger diagnostic or prognostic biomarkers for lung cancer patients, which implies the same meaning that the expression of tissue *microRNAs* significantly alters in cancer patients compared with healthy controls. By subgroup analysis, different treatment to subjects could be an influence to get a quite accurate outcome, which leads to a different survival time of patients. The length of the time for following up the patients may not have an evident difference, this phenomenon might be explained that when lung cancer patients were examined, most of them have been in an advanced stage and had a relatively short survival time, which is also the reason and value of our research.

This meta‐analysis is the first one to study both oncogenic and tumor suppressive *microRNAs* together in affecting the prognosis of *NSCLC* patients. We aimed to get reliable biomarkers that provide valuable information for clinical doctors to carry out the most efficient treatment for *NSCLC* patients and to adjust the treatment strategies. Serum *microRNAs*, having no significant difference with plasma *microRNAs*, tend to have a lower risk compared with *microRNAs* that were from tissue, and this may lead to remind us that special treatment are more needed once oncogenic microRNAs were found in tissue. According to Kentaro's research 22, circulating microRNAs may have less oncogenic genes, and this may be another explanation for its results. Taking the follow‐up time into consideration, there is no evident difference between the timeless and longer than 5 years.

Although our analysis showed *microRNAs* played an important role for *NSCLC* patients’ prognosis in predicting the final outcome, several key limitations could not be ignored. First, we calculated two HRs and collected two *RRs* because of lacking of accurate values in original papers. Those studies in which HR could not be calculated had been removed from analyses, this may reduce some persuasion to its conclusion relatively. Second, the number of the patients included in the study was not large enough to get more accurate results, and the basic clinical characteristics of the patients were also different from each other, which can mainly explain its heterogeneity. Third, the analyses about the *DFS* were really short of, so we could not obtain a relatively correct conclusion about the association between the *microRNAs* and *DFS*. Finally, the number of tumor suppressive *microRNAs* is less than oncogenic *microRNAs* for this study, which may influence the result about protective *microRNAs*.

## Conclusion

In summation, this meta‐analysis demonstrates the function of *microRNAs* in predicting the prognosis of patients with *NSCLC*. Increased tumor suppressive *microRNAs* and decreased oncogenic *microRNAs* are beneficial to advanced *NSCLC* patients by increasing the overall survival, which should be advocated in clinical practice. *NSCLC* patients need more urgent treatment once oncogenic *microRNAs* were found in tissue. In addition, tumor suppressive biomarkers for lung cancer patients needs more researches to strength its persuasion.

## Ethical Approval

This article does not contain any studies with human participants or animals performed by any of the authors.

## Conflict of Interest

The authors have no conflicts of interest to disclose.

## Supporting information


**Table S**1**.** Searching strategies in Pubmed.
**Table S**2**.** Searching strategies in Embase.
**Table S3.** Searching strategies in Web of Science.
**Table S4.** The Newcastle‐Ottawa Scale (*NOS*) used to assess the quality of the 12 studies included in the meta‐analysis.
**Table S5.** Characteristics of the patients included in this meta‐analysis.
**Figure S**1**.** Flow diagram of literatures selected for the meta‐analysis.
**Figure S**2**.** Meta‐analysis of subtotal HRs based on different follow‐up time in predicting the OS of NSCLC patients.
**Figure S**3**.** Meta‐analysis of subtotal HRs based on different treatment in predicting the OS of NSCLC patients.
**Figure S4.** Begger's regression tests for publication bias of OS meta‐analysis.
**Figure S5.** Egger's regression tests for publication bias of OS meta‐ analysis.
