# Supplementary material for: A meta‐analysis: microRNAs’ prognostic function in patients with nonsmall cell lung cancer
Source: Cancer Med. 2017 Aug 15;6(9):2098–105. doi: 10.1002/cam4.1158 (PMC5603832; doi:10.1002/cam4.1158)
Supplement: Supplementary file 1 — Table S1. Searching strategies in Pubmed. Table S2. Searching strategies in Embase. Table S3. Searching strategies in Web of Science. Table S4. The Newcastle‐Ottawa Scale (NOS) used to assess the quality of the 12 studies included in the meta‐analysis. Table S5. Characteristics of the patients included in this meta‐analysis. Figure S1. Flow diagram of literatures selected for the meta‐analysis. Figure S2. Meta‐analysis of subtotal HRs based on different follow‐up time in predicting the OS of NSCLC patients. Figure S3. Meta‐analysis of subtotal HRs based on different treatment in predicting the OS of NSCLC patients. Figure S4. Begger's regression tests for publication bias of OS meta‐analysis. Figure S5. Egger's regression tests for publication bias of OS meta‐ analysis. [file CAM4-6-2098-s001.pdf]

Supplementary table 1 Search strategy for potential references on PubMed

Search	Query	Items found
#14	Search #13 Filters: Humans; English	248
#13	Search #12 Filters: English	333
#12	Search #3 AND #6 AND #11	338
#11	Search #7 OR #8 OR #9 OR #10	1362086
#10	Search HR[Title/Abstract]	187655
#9	Search prognos*[Title/Abstract]	470535
#8	Search survi*[Title/Abstract]	905668
#7	Search kaplan-meier method[Title/Abstract]	12250
#6	Search #4 OR #5	47056
#5	Search microRNA[Title/Abstract]	32460
#4	Search miRNA[Title/Abstract]	29436
#3	Search #1 OR #2	44450
#2	Search *NSCLC*[Title/Abstract]	29191
#1	Search non small cell lung cancer[Title/Abstract]	39501

Supplementary table 2 Search strategy for potential references on EMbase

(http://www.elsevier.com/online -tools/embase)

No.	Query	Results
#13	**#3** AND **#6** AND **#11** AND [english]/lim	**568**
#12	**#3** AND **#6** AND **#11**	**576**
#11	**#7** OR **#8** OR **#9** OR **#10**	**1904166**
#10	**hr**:ab,ti	**311539**
#9	**prognos*:**ab,ti	**676712**
#8	**survi*:**ab,ti	**1237912**
#7	**'kaplan meier method'**:ab,ti	**23353**
#6	**#4** OR **#5**	**62145**
#5	**microrna**:ab,ti	**40422**
#4	**mirna**:ab,ti	**40258**
#3	**#1** OR **#2**	**76173**
#2	**non**:ab,ti AND **small**:ab,ti AND **cell**:ab,ti AND **lung**:ab,ti AND **cancer**:ab,ti	**66562**
#1	***nsclc***:ab,ti	**50272**

Supplementary table 3 Search strategy for potential references on Web of Science and Chinese National Knowledge Infrastructure (http://www.cnki.net/)

检索式		检索结果
# 13	**984**	#11 AND #6 AND #3<br>**精炼依据: 语种:** (ENGLISH)<br>*时间跨度=所有年份,检索语言=自动*
# 12	**1,004**	#11 AND #6 AND #3<br>*时间跨度=所有年份,检索语言=自动*
# 11	**2,686,161**	#10 OR #9 OR #8 OR #7<br>*时间跨度=所有年份,检索语言=自动*
# 10	**310,035**	**主题:** (HR)<br>*时间跨度=所有年份,检索语言=自动*
# 9	**967,212**	**主题:** (prognos*)<br>*时间跨度=所有年份,检索语言=自动*
# 8	**1,772,155**	**主题:** (survi*)<br>*时间跨度=所有年份,检索语言=自动*
# 7	**84,622**	**主题:** (kaplan-meier method)<br>*时间跨度=所有年份,检索语言=自动*
# 6	**82,670**	#5 OR #4<br>*时间跨度=所有年份,检索语言=自动*
# 5	**44,701**	**主题:** (miRNA)<br>*时间跨度=所有年份,检索语言=自动*
# 4	**69,870**	**主题:** (microRNA)<br>*时间跨度=所有年份,检索语言=自动*
# 3	**96,577**	#2 OR #1<br>*时间跨度=所有年份,检索语言=自动*
# 2	**90,216**	**主题:** (non small cell lung cancer)<br>*时间跨度=所有年份,检索语言=自动*
# 1	**49,613**	**主题:** (*NSCLC*)

时间跨度=所有年份,检索语言=自动

Supplementary table 4 The Newcastle-Ottawa Scale (*NOS*) used to assess the quality of the 12 studies included in the meta-analysis

Author (Year)	Selection	Comparability	Outcome
Q.Y .Liu2016	★★	★★	★★★
D. Petriella2015	★★★	★★	★★★
W .Zhao2015	★★	★★	★★★
C.H .Wu2014	★★	★★	★★★
E.H. Cui2012	★★★	★★	★★★
X.G. Liu2011	★★	★★	★★
W.Y .Zhu2011	★★★	★★	★★★
Z.X .Wang2011	★★	★★	★★★
M. K Kim2014	★★★	★★	★★
W.G. Guo 2015	★★	★★	★★★
D.P. Mo2015	★★	★★	★★★
Y.L. Yang2015	★★★	★★	★★★

Supplementary table 5 Characteristics of the patients included in this meta-analysis

Study ID	age		gender		Lymph node metastasis		stage				smoking history		Type of tumor		
	<60	>60	male	female	negative	positive	I	II	III	IV	smokers	nonsmokers	SCC	AC	others
Q.Y .Liu2016	58.49 ± 9.95		137	59	-	-	100			87	130	66	73	115	8
D. Petriella2015	-		21	9	-	-	-		8	22	23	7	11	19	-
W .Zhao2015	-		49	31	-	-	-				-	-	-		
C.H .Wu2014	33	28	38	23	28	33	15	19	27	-	40	14	16	21	24
E.H. Cui2012	56.45+3.4		198	62	-	-	99	101	60	-	176	84	101	117	42
X.G. Liu2011	34	36	56	14	38	32	36	34			46	24	36	34	-
W.Y .Zhu2011	34	36	56	14	38	32	36	34			46	24	36	34	-
Z.X .Wang2011	-		42	46	53	35	47		41		50	38	21	37	30
M. K Kim2014	64(26-77)		57	15	39	33	24	13	25	10	50	22	-		
W.G. Guo 2015	10	15	16	9	9	16	8	17	-	-	16	9	8	12	5
D.P. Mo2015	33	40	53	20	23	50	17	14	20	22	40	33	20	53	-
Y.L. Yang2015	36	38	49	25	43	31	12	28	18	16	-	-	28	46	-

AC: Adenocarcinoma SCC: Squamous cell carcinoma

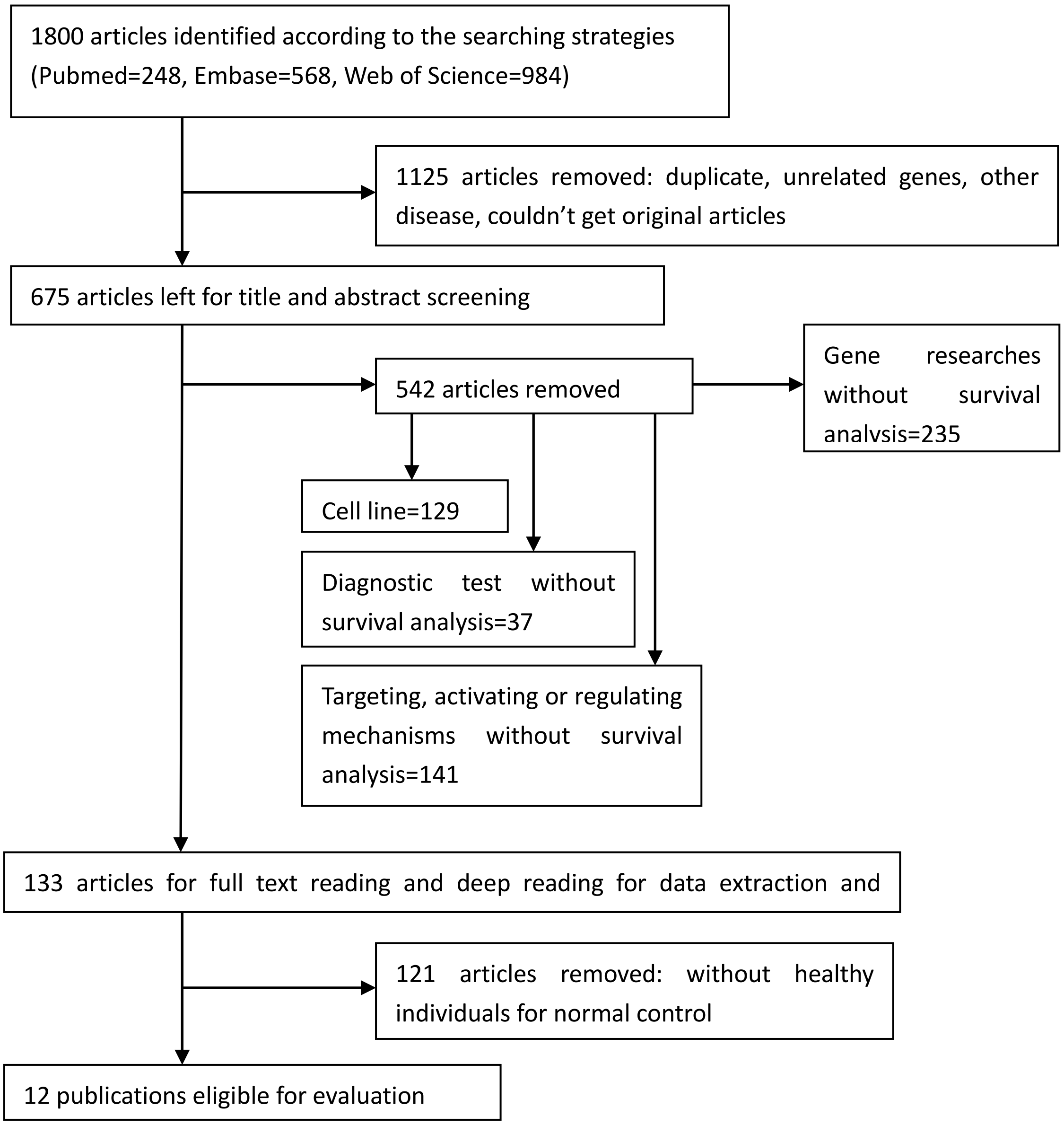

Supplementary Figure 1. Flow diagram of the identification and selection of the studies

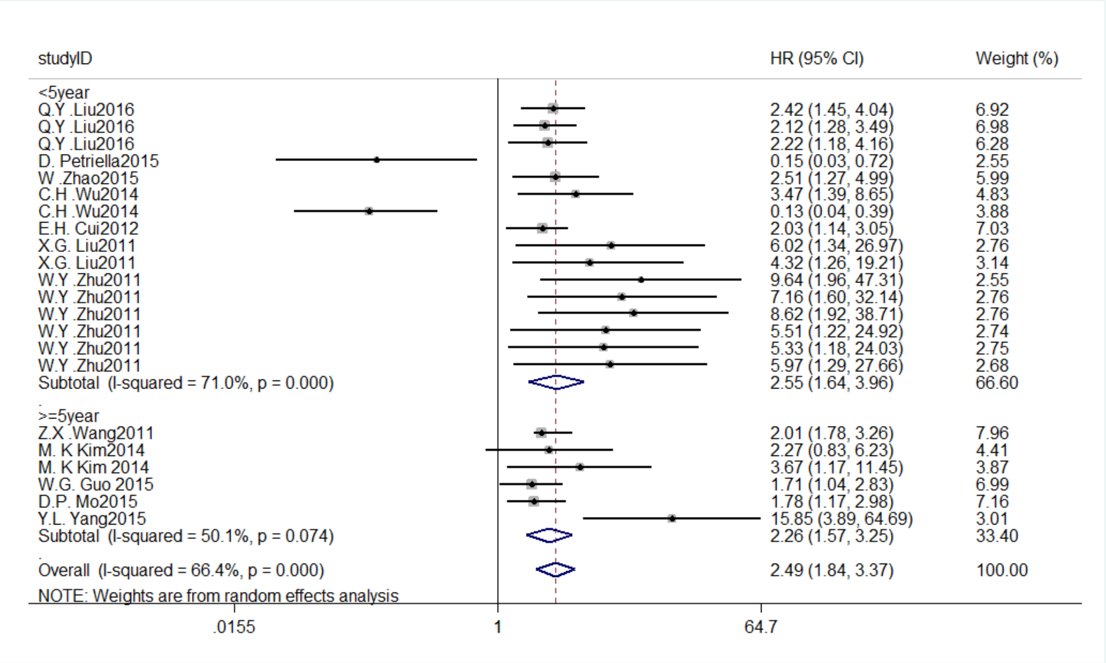

Supplementary Figure 2. Meta-analysis of subtotal HRs based on different follow-up time in predicting the OS of NSCLC patients

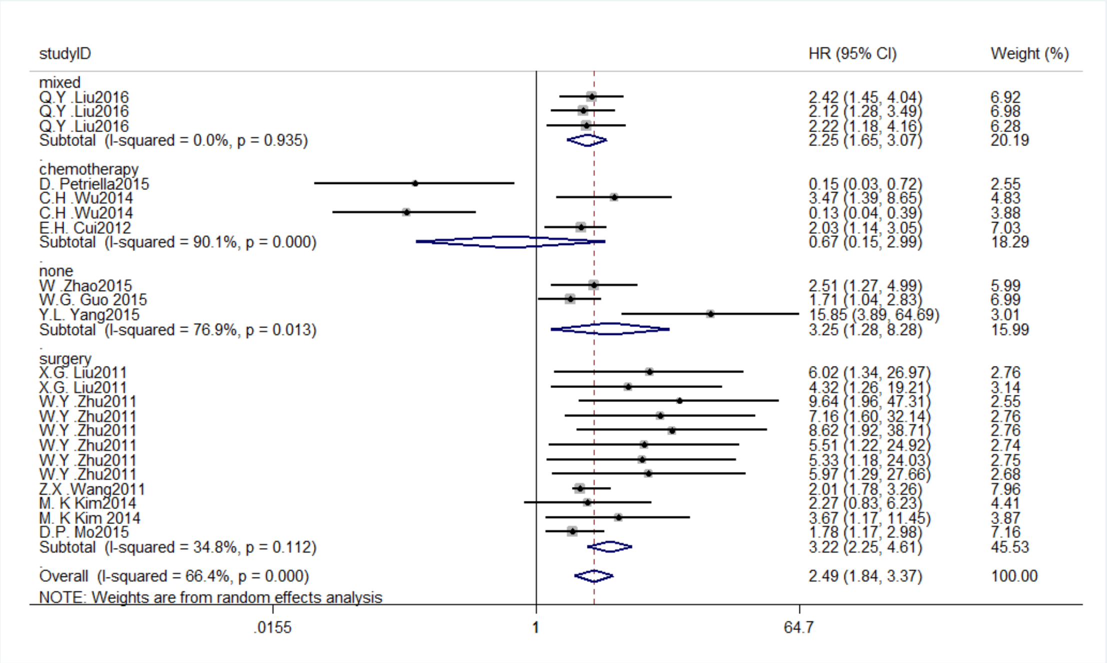

Supplementary Figure 3. Meta-analysis of subtotal HRs based on different treatment in predicting the OS of NSCLC patients

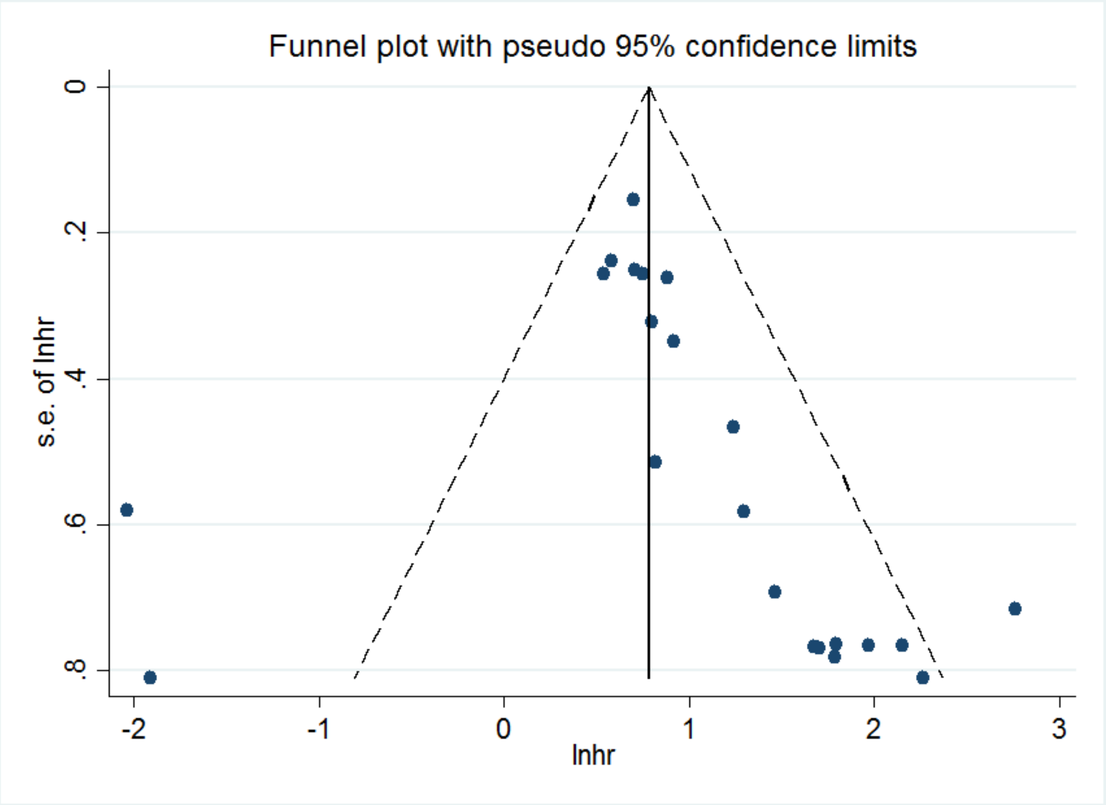

Supplementary Figure 4. Begger’s regression tests for publication bias of OS meta-analysis

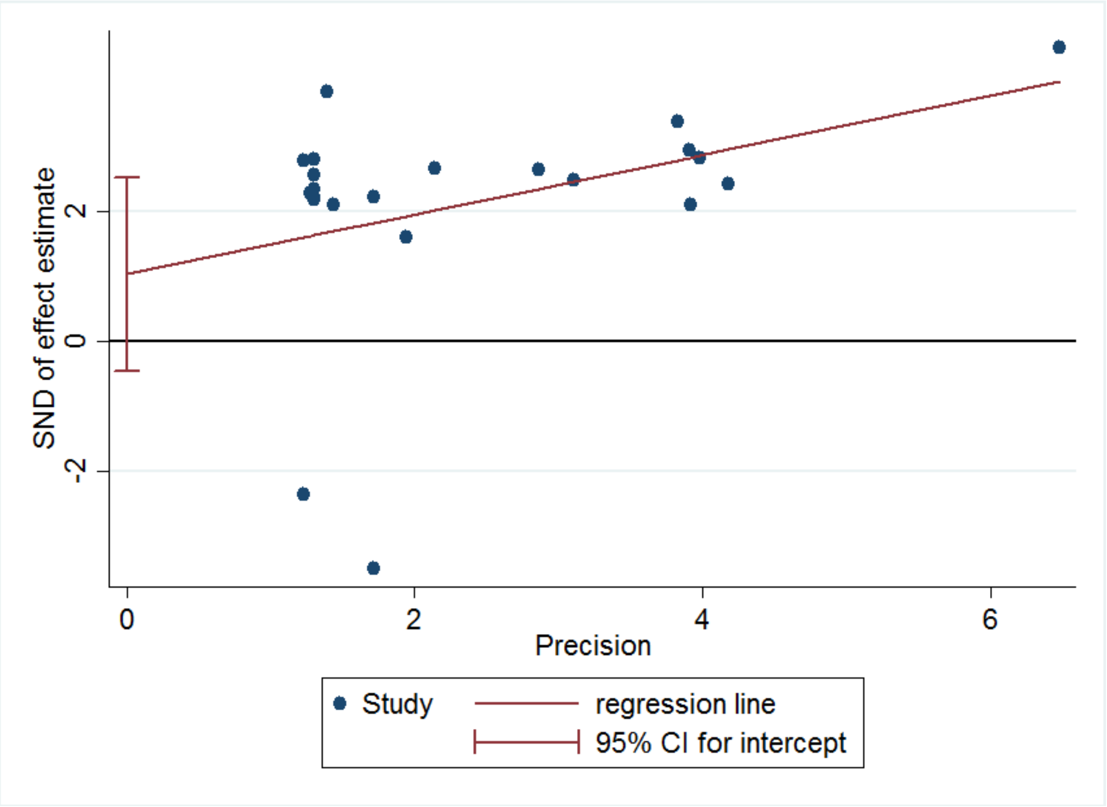

Supplementary Figure 5. Egger's regression tests for publication bias of OS meta-analysis
